# Supplementary material for: Pancreatic Stone Protein as a Biomarker for Sepsis at the Emergency Department of a Large Tertiary Hospital
Source: Pathogens. 2022 May 9;11(5):559. doi: 10.3390/pathogens11050559 (PMC9145478; doi:10.3390/pathogens11050559)
Supplement: Supplementary file 1 [file pathogens-11-00559-s001.zip › pathogens-1703128-supplementary.pdf]

Table S1. MEWS score.

	Points						
	**3**	**2**	**1**	**0**	**1**	**2**	**3**
Systolic blood pressure (mmHg)	<70	71–80	81–100	101–199		≥200	
Heart rate (bpm)		<40	41–50	51–100	101–110	111–129	≥130
Respiratory rate (/min)		<9		9–14	15–20	21–29	≥30
Temperature (°C)		<35		35–38.4		≥38.5	
Neurological score				Alert	Reacting to	Reacting to pain	Unresponsive
